# *Ca*Xyn30B from the solventogenic bacterium *Clostridium acetobutylicum* is a glucuronic acid-dependent endoxylanase

**DOI:** 10.1186/s13104-020-05091-5

**Published:** 2020-06-10

**Authors:** Casey Crooks, Liangkun Long, Franz J. St John

**Affiliations:** 1 Institute for Microbial and Biochemical Technology, Forest Products Laboratory, USDA Forest Service, One Gifford Pinchot Drive, Madison, WI 53726 USA; 2 College of Chemical Engineering, Nanjing Forestry University, Nanjing, 210037 China

**Keywords:** Glycoside hydrolase, Xylanase, GH30, Glucuronoxylan, Bioconversion, Biorefinery

## Abstract

**Objective:**

We previously described the structure and activity of a glycoside hydrolase family 30 subfamily 8 (GH30-8) endoxylanase, *Ca*Xyn30A, from *Clostridium acetobutylicum* which exhibited novel glucuronic acid (GA)-independent activity. Immediately downstream from *Ca*Xyn30A is encoded another GH30-8 enzyme, *Ca*Xyn30B. While *Ca*Xyn30A deviated substantially in the highly conserved β7-α7 and β8-α8 loop regions of the catalytic cleft which are responsible for GA-dependence, *Ca*Xyn30B maintains these conserved subfamily 8 amino acid residues thus predicting canonical GA-dependent activity. In this report, we show that *Ca*Xyn30B functions as a canonical GA-dependent GH30-8 endoxylanase in contrast to its GA-independent neighbor, *Ca*Xyn30A.

**Results:**

A clone expressing the catalytic domain of *Ca*Xyn30B (*Ca*Xyn30B-CD) exhibited GA-dependent endoxylanase activity. Digestion of glucuronoxylan generated a ladder of aldouronate limit products as anticipated for canonical GA-dependent GH30-8 enzymes. Unlike the previously described *Ca*Xyn30A-CD, *Ca*Xyn30B-CD showed no activity on arabinoxylan or the generation of appreciable neutral oligosaccharides from glucuronoxylan substrates. These results are consistent with amino acid sequence comparisons of the catalytic cleft and phylogenetic analysis.

## Introduction

**Fig. 1:**
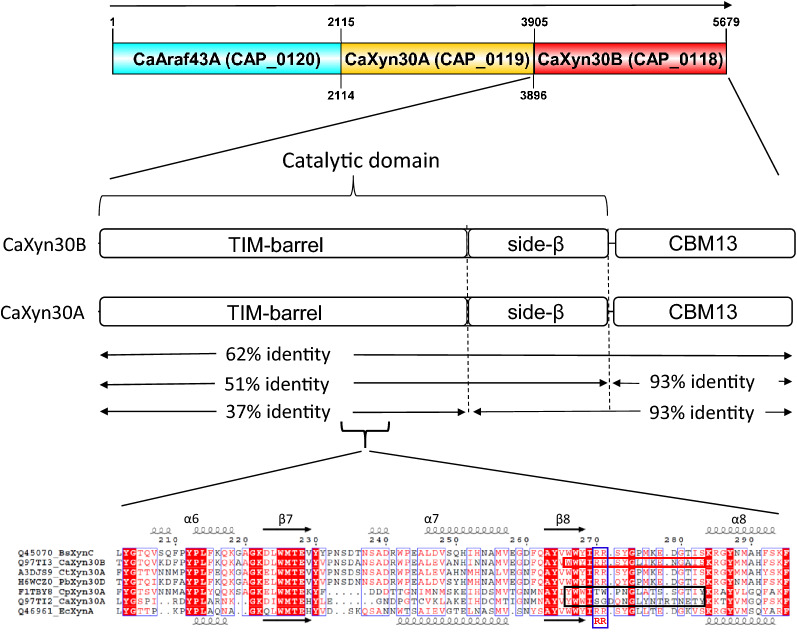
Homology comparison of *Ca*Xyn30B (UniProt Q97TI3) with *Ca*Xyn30A (UniProt Q97TI2) from *C. acetobutylicum* ATCC 824. Gene organization of the predicted operon containing *Ca*Xyn30B and the previously described GA-independent endoxylanase, *Ca*Xyn30A. GenBank locus tag identifiers are shown in parenthesis, the arrow indicates direction of transcription (top). Depiction of C-terminal bias of homology between these linked genes. While the proteins show high homology overall, the homology is heavily weighted towards the non-specificity determining C-terminal regions (middle). Alignment detail comparisons of the β7-α7/β8-α8 catalytic cleft of representative gram-positive GA-dependent GH30-8 endoxylanases from *Bacillus subtilis* (*Bs*XynC), *Ca*Xyn30B (this study), *Clostridium thermocellum* (*Ct*Xyn30A), and *Paenibacillusbarcinonensis* BP-23 (*Pb*Xyn30D), with the characterized GA-independent GH30-8 endoxylanases from *Clostridium papyrosolvens* (*Cp*Xyn30A), the linked *Ca*Xyn30A, and a gram-negative GA-dependent representative from *Erwinia chrysanthemi* (*Ec*XynA) (bottom). The β8-α8 loop region of *Ca*Xyn30B is boxed in red, the corresponding loop region in the GA-independent endoxylanases are boxed in black and the location of the GA coordinating “RR-motif” is boxed in blue and highlighted below. Operon prediction provided by www.microbesonline.org/operons/gnc272562.html

Xylan represents the most abundant form of hemicellulose and consists of β-(1,4)-linked d-xylose units. Effective use of xylan in applications including production of renewable fuels, green chemicals and nutraceuticals would be greatly facilitated by the availability of xylanases with well-defined functions that enable the generation of specific product streams [1, 2]. Depending on the xylan source, the main xylan chain may be decorated with acetyl, α-l-arabinofuranose or 4-O-methyl- α-d-glucuronic acid substitutions. The hydrolysis of xylan by endoxylanases is significantly influenced by the xylan substitution characteristics. For strict endoxylanases, more xylan chain substitutions typically result in lower observed activity. GH30-8 endoxylanases are dual domain enzymes consisting of a (β/α)_8_-barrel containing the catalytic determinants with an obligatory stabilizing side β-sandwich structure tightly associated with the (β/α)_8_-barrel catalytic core through hydrophobic contacts [3–5]. Canonical GH30-8 enzymes exhibit a distinct specificity by requiring the recognition of GA substitutions at the catalytic-2b subsite for hydrolysis [5, 6]. In part, this specificity is provided through a salt bridge interaction between the C6 carboxylate of GA and the guanidinium side-chain of a highly conserved arginine in the catalytic substrate binding cleft [6–8]. This arginine and surrounding motif (RR-motif) in the β8–α8 loop region of these enzymes appears critical in determining the substrate specificity of canonical GA-dependent GH30-8 enzymes (Fig. 1). We recently described a novel GH30-8 from *Clostridium acetobutylicum*, *Ca*Xyn30A (UniProt ID: Q97TI2), that lacks the highly conserved RR-motif and found it exhibited a novel GA-*in*dependent activity [9]. Encoded immediately downstream as part of a predicted three gene operon from the non-canonical, GA-independent, *Ca*Xyn30A is *Ca*Xyn30B (UniProt ID: Q97TI3) (Fig. 1). Phylogenetic and structural predictions and the conserved RR-motif of *Ca*Xyn30B suggest that this enzyme will exhibit canonical GH30-8 GA-dependent endoxylanase activity exemplified by the previously described GH30-8 endoxylanase from *Bacillus subtilis*, *Bs*XynC, or the gram negative representative, *Ec*XynA, from *Erwinia chrysanthemi* [5, 6]. Here we describe the biophysical functional parameters of this genetically linked GH30-8 enzyme and confirm that it functions primarily as a canonical GH30-8 GA-dependent endoxylanase.

## Main text

### Methods

#### Chemicals

All general use chemicals were at least ACS grade. Molecular biology reagents were purchased from New England Biolabs (Ipswich, MA). For xylanase assays beechwood xylan (BX) was obtained from Sigma (St. Louis, MO), wheat arabinoxylan (WAX) and xylohexaose were obtained from Megazyme International (Wicklow, Ireland). Sweetgum wood xylan (SGX) was obtained from the laboratory of Dr. James F. Preston from the University of Florida. The aldotetrauronic acid (GX3) was prepared by digesting BX to completion with the GH10 endoxylanase, *Pb*Xyn10A1CD [10, 11], and purifying using preparative TLC. Using the same TLC solvent system and detection described below for analytical studies, preparative TLC was performed using 20 cm × 20 cm, 500 µm thick silica gel H (no binder) plates. The *Pb*Xyn10A1CD BX hydrolysate was spotted in bands using the BandIT preparative TLC sample applicator from Miles Scientific (Newark, DE). The plate was developed with two ascensions and the sides were removed and developed to guide sample recovery. GX3 migration was identified using GXn standards and was isolated by washing the recovered silica in excess water. This volume was lyophilized, redissolved in water and the volume used in assays was determined empirically by comparison to quantified xylooligosaccharides.

#### Cloning of CaXyn30B

*Ca*Xyn30B (UniProt ID: Q97TI3), encoded on the pSOL1 megaplasmid, was isolated from genomic DNA obtained from *Clostridium acetobutylicum* ATCC 824. PCR amplification of the catalytic domain of *Ca*Xyn30B was performed using primers 5′-ctctaCCATGGcttcaaatgttatggttaatttagcctc-3′ and 5′-cctcaCTCGAGgttgttagtaggctcaaatacc-3′ and cloned into pET28b (Novagen) between the NcoI and XhoI sites using the sites as indicated with uppercase in the primers shown. The resulting construct was sequence verified and encodes a 402 amino acid C-terminal hexahistidine tagged expression product, *Ca*Xyn30B-CD.

#### Protein expression of CaXyn30B-CD

*Ca*Xyn30B-CD was expressed in *E. coli* using a modified auto-induction method derived from Studier et al. [12]. Briefly, the pET28-*Ca*Xyn30B-CD plasmid was transformed into Rosetta 2(DE3) cells and maintained under selection with 50 µg/ml kanamycin and 34 µg/ml chloramphenicol. A single colony was propagated in non-induction media (50 mM Na_2_HPO_4_, 50 mM KH_2_PO_4_, 50 mM NH_4_Cl, 5 mM Na_2_SO_4_, 2 mM MgSO_4_, 0.5 × trace metals mix (Technova), 0.5% glucose, 0.25% sodium aspartate) grown overnight at 37 °C and 250 RPM. A 1:150 inoculum was introduced into auto-induction media (0.5× terrific broth, 50 mM NH_4_Cl, 5 mM Na_2_SO_4_, 2 mM MgSO_4_, 0.5× trace metals mix, 0.25% sodium aspartate) and grown for 40 h at 18 °C, 250 RPM. Expression cultures were harvested, processed, and protein purified as previously described using Ni^2+^ immobilized metal affinity chromatography [11]. The eluted protein was desalted with two passages through Zeba 7 kDa size exclusion spin column (Thermo Fisher Scientific). Protein size was confirmed and purity was empirically estimated at > 95% by SDS-PAGE [13] (not shown).

#### Enzyme assays

Enzyme activity optimization and specific activity was determined using the Nelson’s test [14, 15] as previously described [8]. In *Ca*Xyn30B reactions, BX was used at 10 mg/ml and buffers at 30 mM. Reactions were initiated by addition of 200 ng of *Ca*Xyn30B. For pH optimization the reaction temperature was 40 °C. Buffers included sodium acetate for lower pH range, a sodium acetate/MES mixture for a middle pH range and sodium phosphate for a higher pH range. Following optimum reaction pH determination, optimum reaction temperature was determined using sodium acetate/MES buffer at pH 5.75. Specific activity was determined as described above, except that pH 6.0 acetate buffer was used instead of the acetate/MES two component buffer and the reaction was performed at 50 °C for 10 min. One unit is defined as the amount of enzyme which liberates 1 µmol of reducing terminal per minute.

#### TLC and MALDI-TOF

Hydrolysis reaction products were analyzed by thin layer chromatography (TLC) and Matrix-Assisted Laser Desorption/Ionization Time of Flight Mass Spectrometry (MALDI-TOF MS). TLC and MALDI-TOF MS were performed as previously reported [8, 16]. Briefly, TLC reactions containing 10 mg/ml SGX, 7.5 mg/ml WAX, or 5 mM of the oligomeric substrates (GX3, X4, X5, X6) were digesting in 50 µl volumes (25 µl for GX3) with 20 µg/ml of *Ca*Xyn30B, 28 µg/ml *Bc*XynC or 5 µg/ml *Ca*Xyn30A. *Ca*Xyn30B and *Bs*XynC were incubated at 40 °C in 30 mM sodium acetate pH 6 buffer while *Ca*Xyn30A was incubated using 30 mM sodium acetate pH 4. All TLC reactions were digested for 30 min and then heat inactivated at 95 °C for 10 min and used directly for spotting on Analtech silica G plates spotting 5 µl of the reaction in 1 µl increments under warm air flow to allow drying between applications. Samples for MALDI-TOF were digested overnight using 3 µg/ml in 30 mM pH 6.0 sodium acetate buffer. Samples were heat killed as above and were decationized using Dowex 50WX4 hydrogen form. The sample was added to the MALDI-TOF matrix 2,5-dihydroxybenzoic acid, spotted on the MALDI-TOF plate and dried for analysis. Mass analysis was performed using a Shimadzu MegaTOF at the University of Wisconsin, Biochemistry Instrumentation Facility.

### Results

In a recent publication we characterized a confidently classified GH30 subfamily 8 endoxylanase (*Ca*Xyn30A, Q97TI2) from *C. acetobutylicum* which showed strikingly divergent function by exhibiting GA-*in*dependent endoxylanase activity as evidenced by the processing of WAX, release of neutral oligosaccharides from xylan polymers, and activity on neutral xylooligosaccharides [9]. This was attributed to amino acid sequence changes in the β8-α8 and β7-α7 loop regions of the enzyme. Other previous studies made a distinction between two primary GH30-8 branches [5]. This occurs between those which derive from Gram-positive bacteria versus those from Gram-negative bacteria. Most interestingly, while *Ca*Xyn30A derives from a Gram-positive bacterial host, it is nevertheless of the Gram-negative GH30-8 subtype. The adjacent enzyme, *Ca*Xyn30B (Q97TI3) which is the focus of this report, does not have any significant divergent gram-negative type sequence [5], is of the Gram-positive subtype and is expected to be a canonical functioning glucuronoxylanase. Interestingly, primary sequence analysis comparisons of *Ca*Xyn30B with *Ca*Xyn30A show that overall sequence homology is very high at about 62% identity for the mature protein (Fig. 1). This is unexpected considering their separate grouping into the Gram-type distinguished GH30-8s [5], which typically have identities less than 40%. More detailed analysis reveals that the C-terminal carbohydrate binding module (CBM, CBM13) which both enzymes share is a primary source of the high identities (i.e. 93% identify for this separate domain), such that exclusion of the CBM13 module results in a 51% identity for the remaining protein. This high homology also extends into the catalytic domain stabilizing side beta-structure. Comparisons of the sequence representing only the specificity determining (β/α)_8_ TIM-barrel domain of the GH30 enzyme [4, 5, 17] yields a lower level of sequence identity of 37%, as expected for enzymes of different Gram-type grouping.

**Fig. 2:**
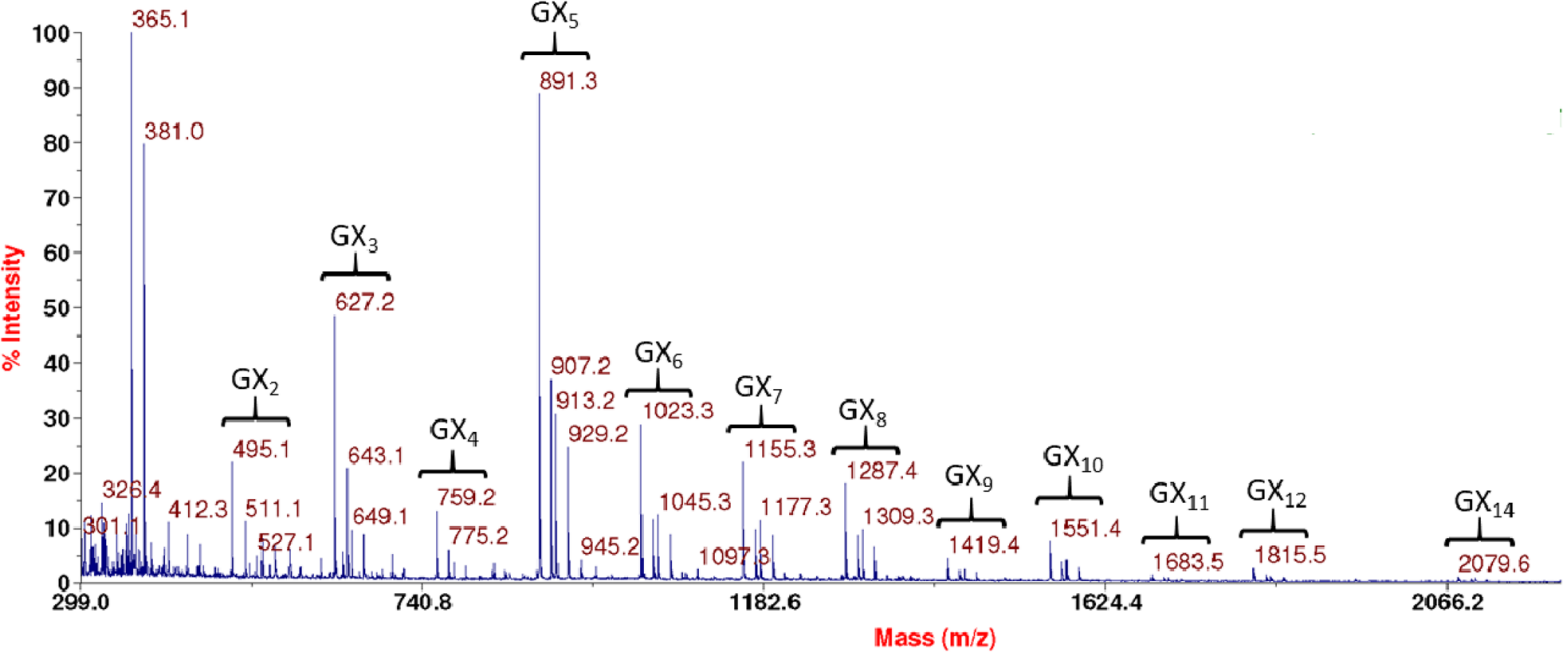
MALDI-TOF results identify the anticipated series of aldouronates each which differs by a single anhydroxylose (132 Da) in mass. The primary peak for each cluster is the single sodium adduct, [GXn-Na]+

**Fig. 3:**
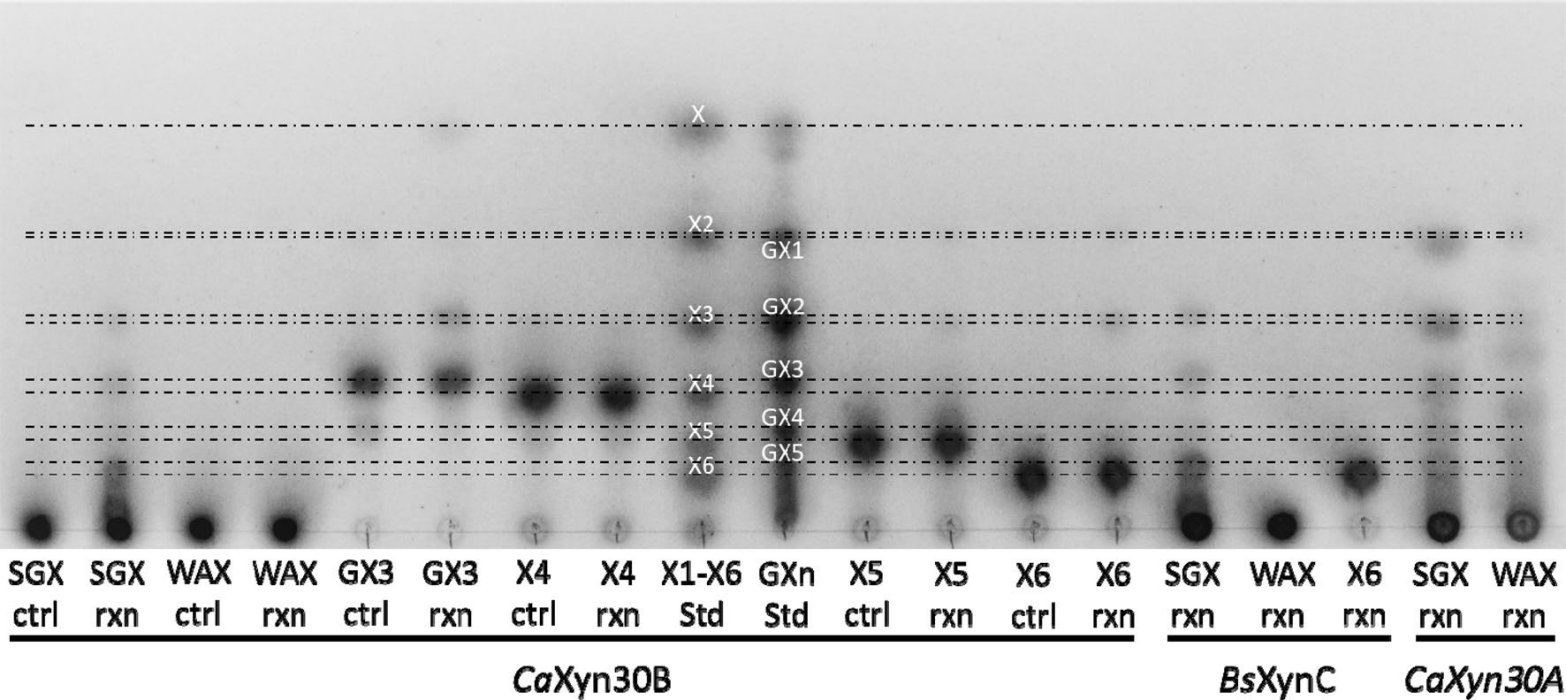
TLC of model xylan and xylooligosaccharides digestions by *Ca*Xyn30B, the GA-dependent endoxylanase, *Bs*XynC, and the previously described GA-independent endoxylanase, *Ca*Xyn30A. From left to right glucuronoxylan (sweetgum, SGX), arabinoxylan (wheat, WAX), aldotetrauronic acid (GX3), aldotriuronic acid (GX2), xylotetraose (X4), xylose to xylohexaose standards (X1-X6), aldouronate standards (GXn), xylopentaose (X5) and xylohexaose (X6)

*Ca*Xyn30B-CD demonstrated GA-dependent activity on xylan substrates. MADLI-TOF MS analysis of glucuronoxylan digested by *Ca*Xyn30B liberated aldouronates as predicted for GA-dependent GH30-8 enzymes and lacked the accumulation of appreciable amounts of neutral xylooligosaccharides (Fig. 2). TLC analysis of digestion of model xylans showed *Ca*Xyn30B to be active on glucuronoxylan (SGX) but not arabinoxylan (WAX), similar to that observed for the defined GA-dependent endoxylanase, *Bs*XynC, but unlike that observed for the previously described GA-independent enzyme, *Ca*Xyn30A, which also showed activity on arabinoxylan (Fig. 3). Digestion of GX3 yielded GX2 plus xylose, while digestion of xylotetraose showed no detectable activity, confirming the coordination of glucuronic acid in the catalytic-2b subsite (Fig. 3) [5, 8]. More activity than expected was observed in the hydrolysis of xylohexaose by *Ca*Xyn30B (Fig. 2). Previous reports of the hydrolysis rate difference between polymeric glucuronoxylan and neural xylooligosaccharides (typically exemplified by X6) were estimated at three orders lower for the unsubstituted linear xylooligosaccharide [6, 18, 19]. From our studies we estimate that *Ca*Xyn30B while still being predominantly GA-dependent, has a greater ability to hydrolyze neutral xylooligosaccharides then previously described GH30-8 GA-dependent endoxylanases. From Nelson’s tests studies using X6 as substrate we estimate that *Ca*Xyn30B hydrolyzes X6 only 2-orders lower than a glucuronoxylan. Nevertheless, these results all confirm the anticipated function *Ca*Xyn30B as a GA-dependent GH30-8 endoxylanase. The pH optimum of *Ca*Xyn30B-CD was determined under three buffer systems spanning pH 3.5–8.0 and found to be pH 5.75 (Additional file 1) in contrast to the linked *Ca*Xyn30A which had pH optimum of 4.0 [9]. Temperature optimum was determined at pH 5.75 and found to be 55 °C for a 10 min reaction. The specific activity of *Ca*Xyn30B measured on BX was approximately 14 U/mg *Ca*Xyn30B (data not shown).

### Discussion

*Ca*Xyn30B is 62% identical to the genetically adjacent previously described endoxylanase, *Ca*Xyn30A. Such high degree of homology would suggest conserved functional specificity but the homology is asymmetrically placed. Specifically, the C-terminal Ricin-like CBM13 domain is 93% identical while that of the catalytic domain shows 51% identity. Moreover, identity in the catalytic domain is strongest in the stabilizing side beta-structure that when virtually removed reduces identity of the specificity determining regions of the core TIM-barrel of the catalytic domain to 37%. It is only with consideration of conservation within the β7-α7 and β8-α8 loop regions defining the catalytic cleft including conservation of the RR-motif that one would anticipate the function of *Ca*Xyn30B to more closely resemble that of the previously described GA-dependent GH30-8 enzyme from *B. subtilis*, *Bs*XynC, than that of the recently described *Ca*Xyn30A.

Such high homology, and it being observed at the nucleic acid level (not shown), is suggestive of a gene duplication event with subsequent diversion in the specificity determining regions. Similarly the high conservation of the CBM13 module suggests similar substrate association albeit with different catalytic specificities. The relative contribution of these two linked enzymes in xylan depolymerization requires further study.

## Limitations

*Ca*Xyn30B was tested on a limited number of substrates and conditions were evaluated and the potential for auxiliary activity in addition to the observed GA-dependent endoxylanase cannot be ruled out.

## Supplementary information

**Additional file 1.** Optimum reaction conditions determination for *Ca*Xyn30B. A) The dependence of activity on pH showing three overlapping alternative buffer compositions. B) Optimum reaction temperature determined for a 10 min reaction period.

## Data Availability

Any supporting data or materials are available from the corresponding author upon request.

## Abbreviations

Ca: *Clostridium acetobutylicum*
GH: Glycosyl hydrolase
GA: Glucuronic acid
CBM: Carbohydrate binding module
*Bs*: *Bacillus subtilis*
WAX: Wheat arabinoxylan
BX: Beechwood xylan
SGX: Sweetgum xylan
*Ct*: *Clostridium thermocellum*
*Cp*: *Clostridium papyrosolvens*
*Ec*: *Erwinia chrysanthemi*
*Pb*: *Paenibacillus*
GX3: Aldotetrauronic acid
