# Supplementary figures and images for: CaXyn30B from the solventogenic bacterium Clostridium acetobutylicum is a glucuronic acid-dependent endoxylanase
Source: BMC Res Notes. 2020 Jun 10;13:281. doi: 10.1186/s13104-020-05091-5 (PMC7285738; doi:10.1186/s13104-020-05091-5)

## Slide 1
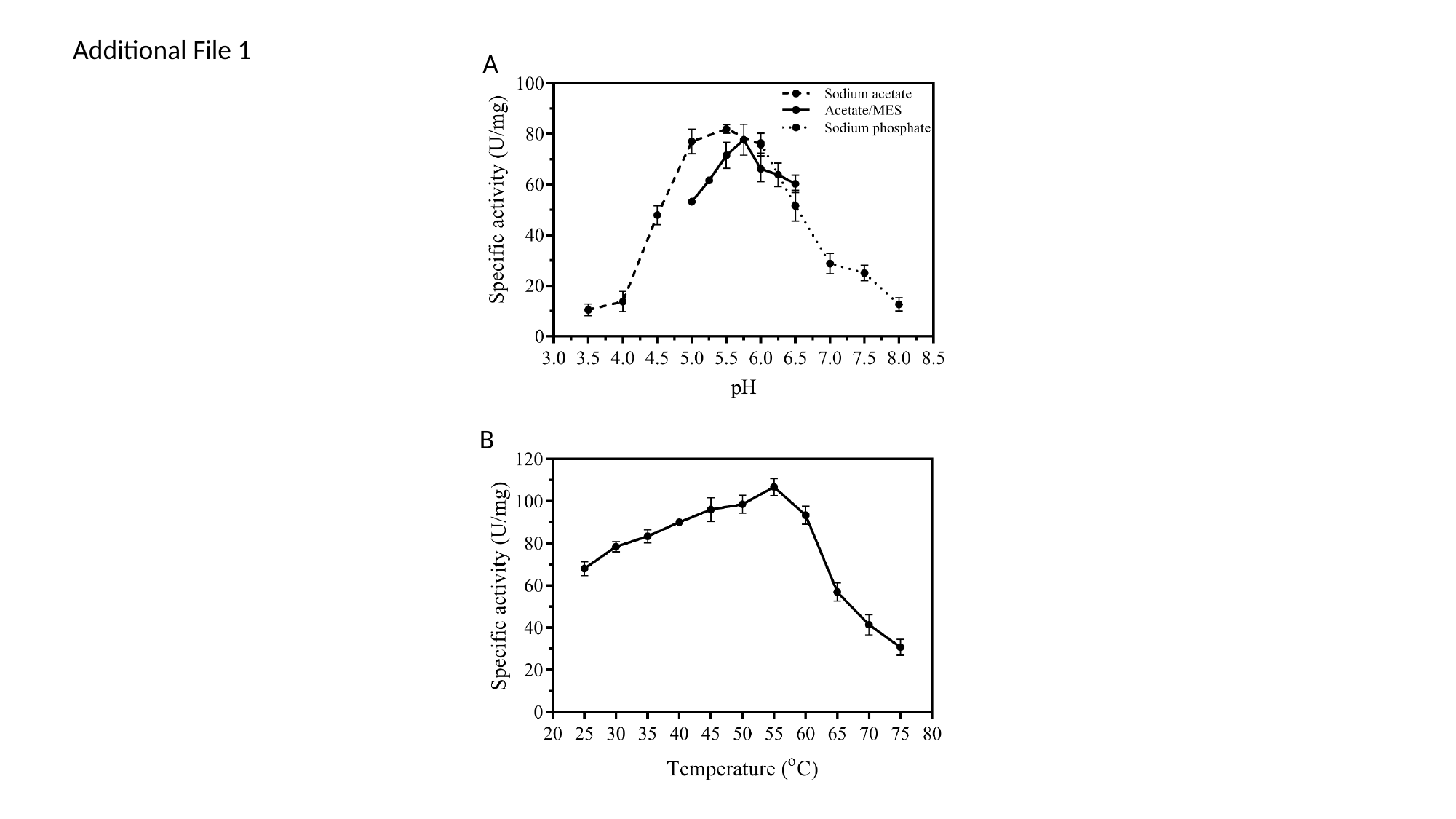

Additional File 1
A
B

Supplement: Supplementary file 1 — Additional file 1. Optimum reaction conditions determination for CaXyn30B. A) The dependence of activity on pH showing three overlapping alternative buffer compositions. B) Optimum reaction temperature determined for a 10 min reaction period. [file 13104_2020_5091_MOESM1_ESM.pptx]
